# Spatial Heterogeneity in Fishing Creates *de facto* Refugia for Endangered Celtic Sea Elasmobranchs

**DOI:** 10.1371/journal.pone.0049307

**Published:** 2012-11-14

**Authors:** Samuel Shephard, Hans Gerritsen, Michel J. Kaiser, David G. Reid

**Affiliations:** 1 Marine and Freshwater Research Centre, Galway-Mayo Institute of Technology, Galway, Ireland; 2 School of Biological Sciences, Queen's University Belfast, Belfast, United Kingdom; 3 Marine Institute, Rinville, Oranmore, Co. Galway, Ireland; 4 School of Ocean Sciences, Bangor University, Anglesey, United Kingdom; Institute of Marine Research, Norway

## Abstract

The life history characteristics of some elasmobranchs make them particularly vulnerable to fishing mortality; about a third of all species are listed by the IUCN as Threatened or Near Threatened. Marine Protected Areas (MPAs) have been suggested as a tool for conservation of elasmobranchs, but they are likely to be effective only if such populations respond to fishing impacts at spatial-scales corresponding to MPA size. Using the example of the Celtic Sea, we modelled elasmobranch biomass (kg h^−1^) in fisheries-independent survey hauls as a function of environmental variables and ‘local’ (within 20 km radius) fishing effort (h y^−1^) recorded from Vessel Monitoring Systems data. Model selection using AIC suggested strongest support for linear mixed effects models in which the variables (i) fishing effort, (ii) geographic location and (iii) demersal fish assemblage had approximately equal importance in explaining elasmobranch biomass. In the eastern Celtic Sea, sampling sites that occurred in the lowest 10% of the observed fishing effort range recorded 10 species of elasmobranch including the critically endangered *Dipturus* spp. The most intensely fished 10% of sites had only three elasmobranch species, with two IUCN listed as Least Concern. Our results suggest that stable spatial heterogeneity in fishing effort creates *de facto* refugia for elasmobranchs in the Celtic Sea. However, changes in the present fisheries management regime could impair the refuge effect by changing fisher's behaviour and displacing effort into these areas.

## Introduction

An emerging requirement of the Ecosystem Approach to Fisheries Management (EAFM) is to understand the spatial scales at which the ecological impacts of fishing operate [1], [2]. Fish communities typically are not homogeneous; structure and composition can vary in space as a function of environmental variables such as habitat and benthic community composition [3], [4], and these patterns of spatial variation can remain consistent over time [5]. Such environmentally driven spatial heterogeneity or ‘patchiness’ in the marine fish community can be reflected in regional variation in size-structure [6]. However, statistical modelling of a metric of size-structure and species composition (the Large Fish Indicator [7]) in the Celtic Sea suggests that the fish community can also vary in space with ‘local’ (within 20–40 km radius) fishing intensity [8]. This fishing effect on spatial size-structure likely occurs because of temporal stability in the regional distribution of fishing effort [9], [10] relative to environment and habitat characteristics (e.g., substratum, [11]). Such stability may reveal time-lagged Pressure-State relationships between a local effort regime and the fish community it affects. In this context, fishing impacts on the seabed (e.g., [12]) and on target communities [1], [13] can be spatially discrete. Correct knowledge of such fishing impacts is critical to the use of spatial management measures (e.g., Marine Protected Areas, MPAs) in conservation and recovery of exploited communities [14]. In particular, by improving our understanding of the appropriate spatial scales at which MPAs might have benefits for species with different life history.

Fishing-induced curtailment of fish community size-structure (e.g., [15], [16]) reflects changes in fish community species composition and evenness [17]. This change typically comprises loss of larger body-sized species having life history traits including slow growth, late age at maturity and low fecundity. These characteristics often render populations particularly vulnerable to incidental [18] or target mortality [19], [20]. A group exemplifying ‘slow’ life history is the elasmobranchs, i.e., sharks, rays and chimaeras, which have among the most complex reproductive strategies of all fishes [21]. In the North Atlantic, relatively few elasmobranch species are targeted commercially (e.g., [22]), but many are known to be vulnerable to fishing (e.g., [23], [24]). Some species of elasmobranchs may even have been extirpated in heavily exploited regions, like the North Sea [25], [26]. In a specific example, common skate *Dipturus batis* was already very rare in the Irish Sea by 1981 [27] and (now classified as two separate species: *Dipturus intermedia* and *D. flossada*) has been listed by the IUCN as Critically Endangered [28].

If fish community size-structure and species composition change in space with environment and fishing intensity, then heterogeneity in distribution and abundance of vulnerable elasmobranchs can be expected. Rogers *et al*. [29] note that current elasmobranch abundance is lowest in the most heavily fished (south-eastern) part of the North Sea, although previously such species were common in this area [30]. Greenstreet *et al*. [31] also observed that demersal fish species diversity has declined in those areas of the North Sea showing greatest fishing effort, with the decline reflecting loss of species such as the globally ‘Vulnerable’ (IUCN) spiny dogfish *Squalus acanthius*. In contrast, Walker & Heessen [23] speculated that areas in the North Sea that are difficult to access with towed gear could become refugia for elasmobranch populations. If such areas of low fishing intensity do act as refugia, this may create opportunities for informed spatial management. There is evidence that formal MPAs can contribute to conservation and management of elasmobranchs [32], [33], although this is strongly contingent on movement patterns [34], which can vary with environmental conditions [35]. For an MPA to succeed, elasmobranch abundance would have to respond to ‘local’ fishing intensity at a scale expedient to realistic (socio-economically acceptable) MPA size [36]. Some modelling studies suggest that temperate MPAs should encompass around 80% of a fish species range, and thus to be successful MPA size must increase with assumed species mobility [37]. However, meta-analysis suggests that temperate protected areas (<100 km^2^) are associated with positive responses in the abundance and biomass of some fish species, although often this coincides with strong habitat association within the MPA boundary [38], [39]. Given the critical conservation status and growing public profile of elasmobranchs, it is important to understand the spatial scale at which MPAs might be effective tools to conserve populations.

The Celtic Sea retains some of the largest remaining populations of many NE Atlantic elasmobranch species [29], including the critically endangered *D. intermedia* and *D. flossada*. In the current paper, we combined fisheries-independent survey data and fine-scale fishing effort (Vessel Monitoring Systems, VMS) data from the Celtic Sea with several key environmental descriptors. The objective was to establish whether spatial heterogeneity in fishing effort can lead to a temporally stable mosaic of fished and unfished areas that would generate *de facto* refugia resulting in local changes in biomass and species composition of elasmobranchs. *De facto* refugia (sensu [40]) are here considered to be areas without formal restrictions on the spatial allocation of fishing effort, but where there are natural obstacles (e.g., rough seabed or distance from port) that act to minimize actual fishing activity. Such refugia may represent sites where establishment of formal MPAs would result in minimal fishing effort displacement and therefore would be good candidate areas for such management interventions.

## Methods

In studies of spatial or temporal variation in fish abundance, standardised catch rate (e.g., Catch Per Unit of Effort, CPUE) is often used. Standardised CPUE accounts for variation in abundance or biomass due to environmental or other factors (see [41] for a review). In the current study, linear mixed effects models that included environmental variables were used to test for an effect of local fishing effort regime (hours fishing per year, h y^−1^) on biomass of elasmobranchs caught per hour of survey trawl sampling (kg h^−1^) in the Celtic Sea.

### Ecological data

**Figure 1:**
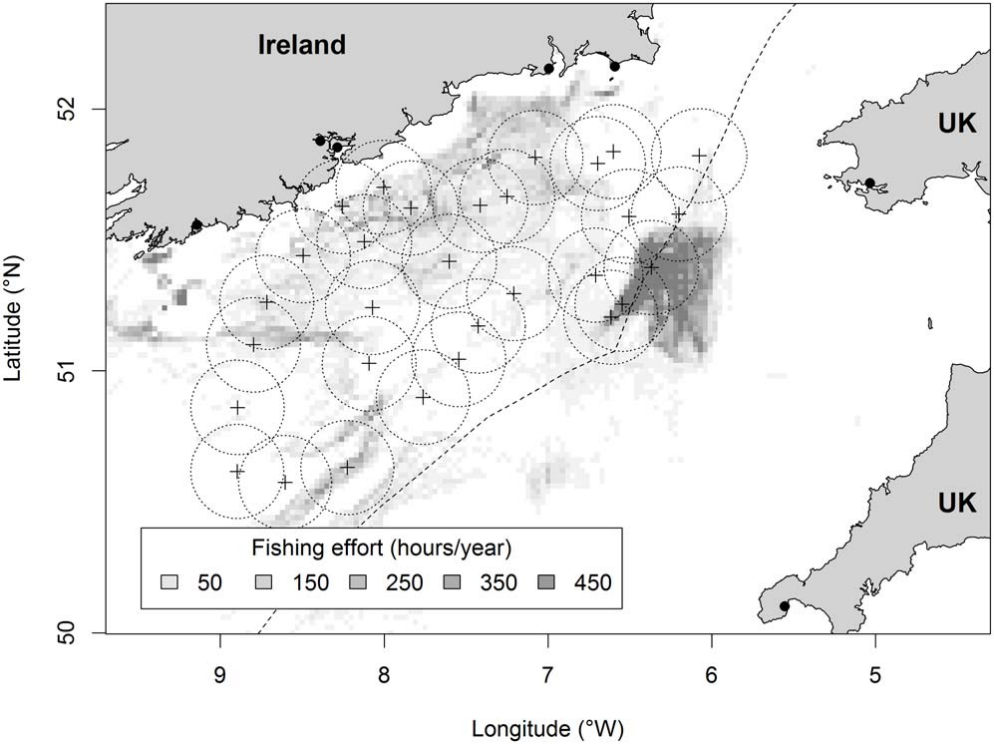
Location of IGFS survey hauls in 2007 with associated 20 km circles; other study years have similar sample distribution. Fishing effort is shown as a background, where increasing effort is represented as darker shading; note that effort data outside the Irish EEZ is incomplete. Black dots indicate main fishing ports. The border between UK and Irish Exclusive Economic Zones (EEZ) is shown.

The Irish Groundfish Survey (IGFS) is a standardized bottom-trawl survey that includes the Celtic Sea (Figure 1), and has occurred in late autumn since 1997. The Irish Marine Institute operate the survey following standard International Bottom Trawl Survey (IBTS) protocol. Sampling gear is a Grande Ouverture Verticale (GOV) trawl fitted with a 20 mm codend liner. In a given year, trawl samples (approx. 30 min duration) are collected at sites randomly selected from a pool of around 100 fixed sampling stations (‘Prime Stations’). All fish captured are identified to species and measured (total length; L).

Using IGFS survey data (2006–2011), catch numbers at length were converted to weight (W) at length using weight at length relationships (W = αL^β^), where the parameters α and β were obtained by direct analysis (common species) or from FishBase (www.fishbase.org). Catch weight at each length class of each demersal fish species in each trawl sample (haul) was then converted to a density (kg h^−1^) by dividing by the precise trawl duration. Elasmobranch species richness (total number of species) and biomass density (kg h^−1^) was then calculated for each survey haul.

**Figure 2:**
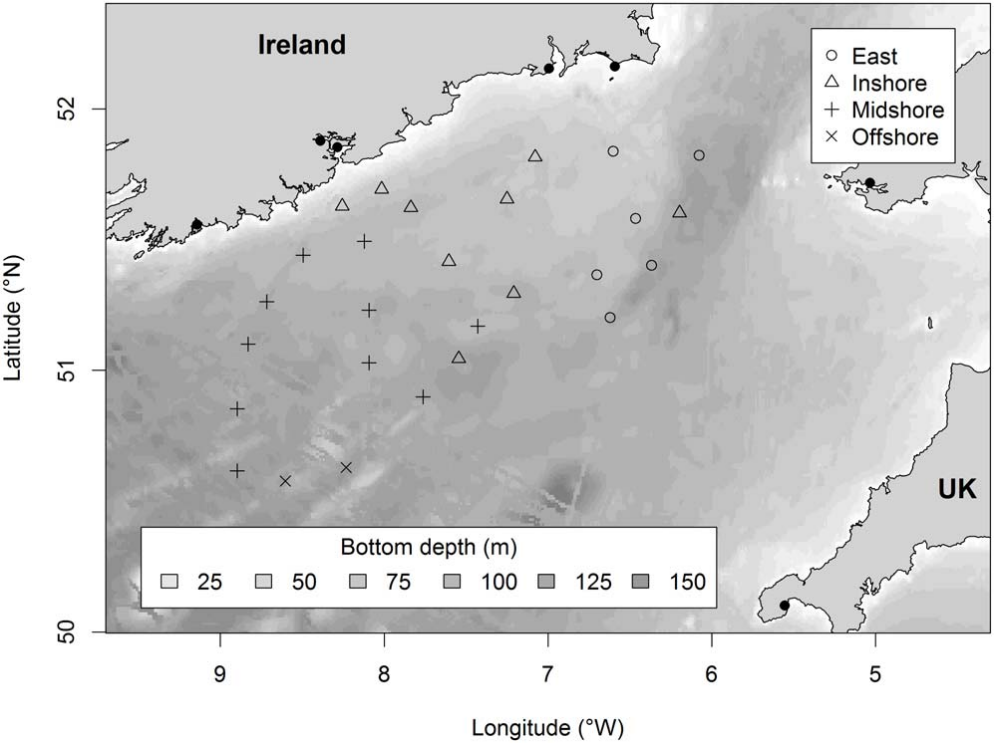
Environmental variables included in models of elasmobranch abundance in the Celtic Sea: Depth, Location (latitude + longitude) and Fish assemblage region (East, Inshore, Midshore, Offshore). Black dots indicate main fishing ports.

In any spatial investigation of the fish community it is necessary to account for biogeography [29]. All stations were allocated to a Celtic Sea biogeographic sub-region based on ‘similar’ [42] demersal fish species composition (henceforth ‘fish assemblage region’) (Figure 2). This factorial variable (having four classes, East, Onshore, Midshore, Offshore) was derived from root-transformed species abundance data from the IGFS. A resemblance matrix was generated using the Bray-Curtis index of similarity, creating a dendrogram using the group-average linkage clustering method and then followed by a SIMPROF test [42] to define clusters *a posteriori* that were significantly (*P*<0.05) different [9]. In the study region (as in the North Sea, [4], [5]) demersal fish assemblage was related to seabed substratum but may also integrate the effects on fish community structure of associated oceanographic variables, especially depth [43]. Each sampling station was also allocated to a substratum class (gravel, sand or mud) using maps available on the Mapping European Seabed Habitats (MESH) website (www.searchmesh.net). Because of differences in the fish community between the shallower eastern area of the Celtic Sea and the deeper western shelf (e.g., [44], [45]), depth (m) and location (longitude + latitude) were also modelled as candidate explanatory variables of relative elasmobranch biomass.

### Fishing effort data

International fishing effort was derived from Vessel Monitoring System (VMS) records (2006–2011) for the area of the Celtic Sea within the Irish Exclusive Economic Zone (EEZ) (Figure 1). VMS transmits the position and speed of fishing vessels at least every 2 hours. All demersal gears (otter and beam trawls and scallop dredges) were included and speed criteria were applied to distinguish fishing activity from steaming and other non-fishing activity. Using records from on-board observers, Gerritsen & Lordan [46] found that for otter bottom trawlers, vessel speeds between 1.5 and 4.5 knots correctly identified fishing activity in 88% of cases. Each VMS record where the vessels were deemed to be fishing was allocated an effort value that was equal to the time interval between successive VMS records (generally 2 hours). For each IGFS sampling station, the value used for analysis was summed annual fishing effort (h y^−1^) within a 20 km radius circle from the survey haul midpoint (Figure 1). International VMS data were only available for survey stations within the Irish EEZ. However, some circles extended outside this national boundary and/or onto land. In these cases, effort was corrected for the area of each circle for which data were available by dividing recorded values by the proportion of each circle comprising sea within the EEZ. Only stations where >50% of the area of the 20 km circle was sea and within the Irish EEZ were used.

### Analysis

**Figure 3:**
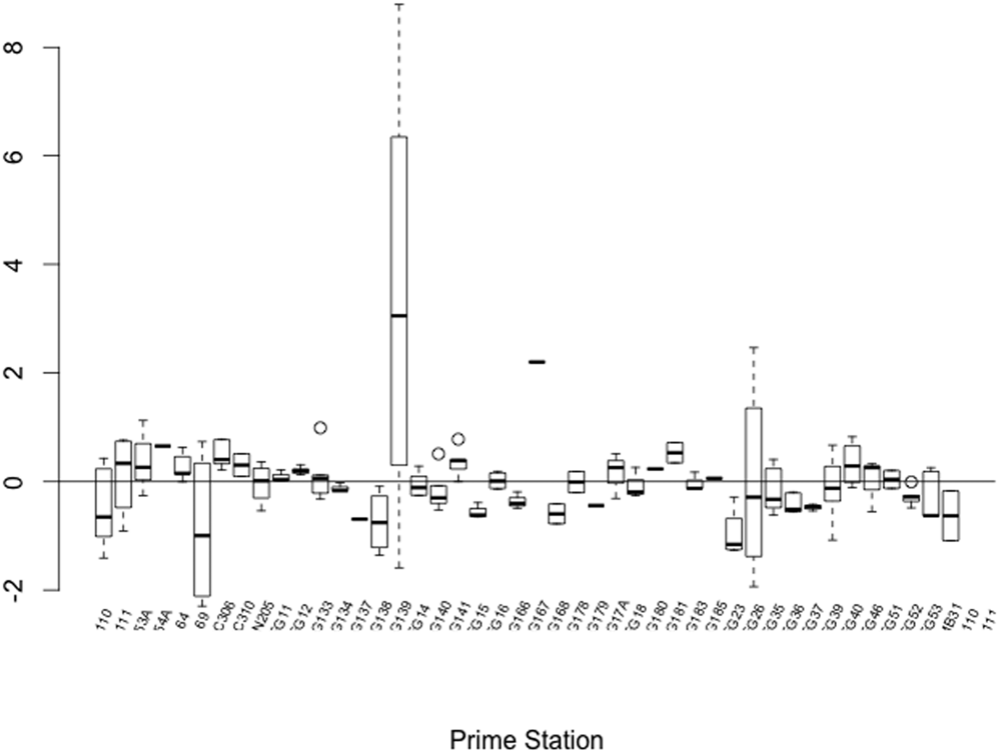
Boxplot of elasmobranch abundance by survey Prime Station. Values are residuals from the ‘best’ (lowest AIC) linear model.

The effect of fishing effort (h y^−1^) on elasmobranch biomass (kg h^−1^) by survey haul was estimated using models that accounted for environmental variables. Model selection was conducted in an information theory context using AIC. The full starting model included: Fish assemblage region (Assemblage), Substratum, Depth (m), sampling location (Location: latitude + longitude) and interactions. A preliminary comparison indicated that a linear model had lower AIC than a (nonlinear) GAM and hence further analysis focused on linear models. Boxplots of model residuals showed variation in elasmobranch biomass by Prime Station (Figure 3), and so a random effect of Prime Station was included. In order to allow direct comparison of model coefficients, numerical variables were standardised such that mean = 0 and variance = 1. The ‘best’ final model (lowest AIC) had the following form:
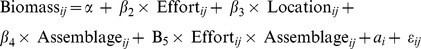



Where: Biomass*_ij_* is elasmobranch biomass (kg h^−1^) for observation (haul) *j* at Prime Station *i* and *a_i_* is the random effect of Prime Station. Residual distributions suggested non-heterogeneity so a structure was added that allowed variance to change with location; this resulted in acceptable residuals. A spline correlogram of model residuals against location (latitude and longitude) showed no spatial autocorrelation.

This statistical modelling indicated a distinct area in the NE Celtic Sea where minimal fishing effort was combined with greater biomass and species richness of elasmobranchs. We hypothesised that this *de facto* refuge developed because fishermen avoid the area for one or more of the following reasons:

1. The catch (landings per unit effort, LPUE) of target species is relatively low in this area.
2. The relative cost of fishing this area, measured as distance from nearest port, is high.
3. The risk of losing gear is unacceptably high due to rough or unpredictable seabed conditions.

Data were not available to support a robust quantitative analysis of this question, so a qualitative approach was taken involving mapping and informal questioning of fishermen who operate around the area.

## Results

**Figure 4:**
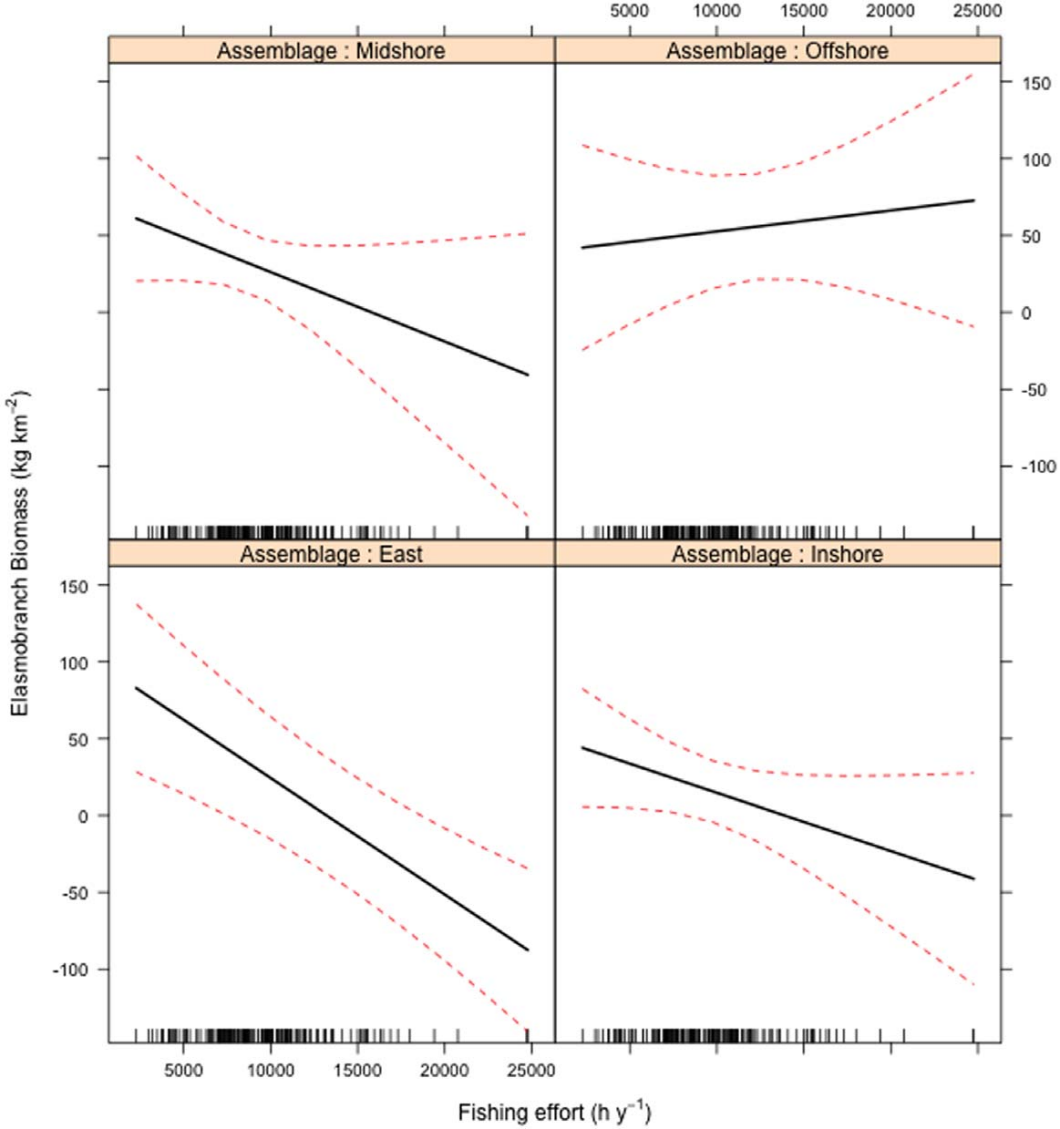
Display of modelled effects on elasmobranch biomass (kg h^−1^): fishing effort (h y^−1^) by demersal fish assemblage region (East, Inshore, Midshore, Offshore).

**Figure 5:**
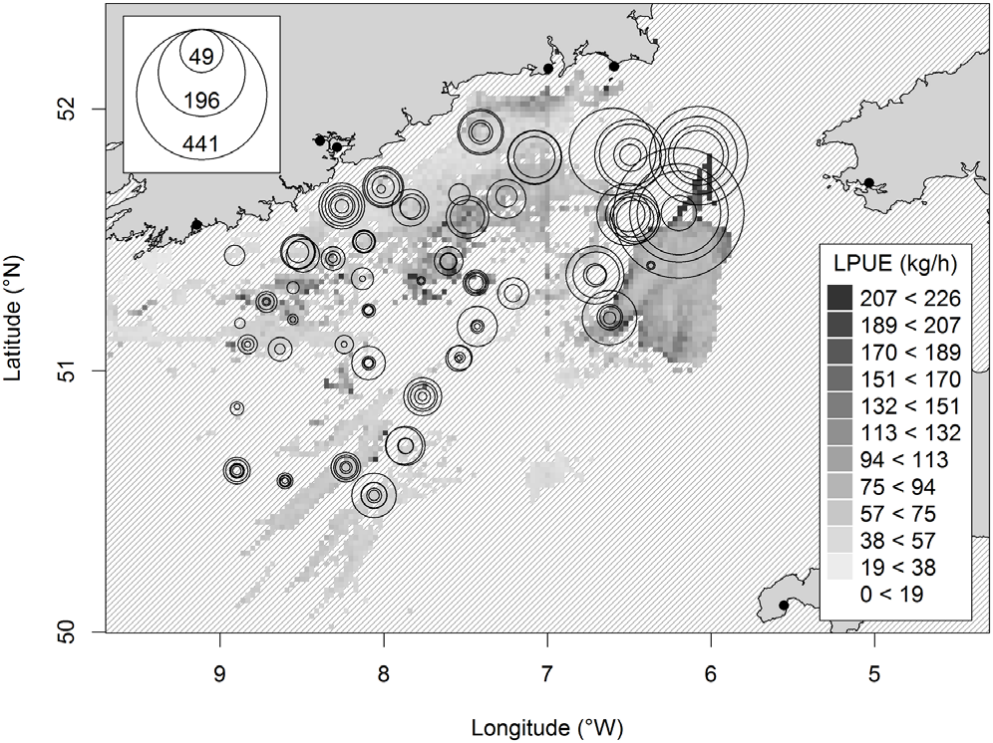
Landings Per Unit of Effort (LPUE) of all commercial species by Irish fishing vessels (2006–2011). The hashed area indicates insufficient data for LPUE estimates. Locations of all IGFS trawl samples used in the current study are shown. Standardized survey elasmobranch biomass (kg h^−1^) by sampling year is illustrated by the size of the bubbles. The legend shows reference bubble sizes with associated biomass values. Black dots indicate main fishing ports.

Model coefficients indicated a negative effect of fishing effort on Celtic Sea elasmobranch biomass. There was also an effect on elasmobranch biomass of fish assemblage region, and an interaction between effort and assemblage region with the strongest effort effect across the ‘East’ region (Figure 4) where greatest elasmobranch biomass was observed (Figure 5). In addition, there was a positive effect on elasmobranch biomass of location (latitude + longitude), with greatest biomass in the NE Celtic Sea. Fishing effort, location and fish assemblage region had approximately equal importance as explanatory variables in the final model (Table 1).

**Table 1:** Coefficients for a model relating standardised elasmobranch biomass (kg h^−1^) in Celtic Sea survey hauls to annual fishing effort (h y^−1^) (2006–2011) within 20 km radius.

Variable	Value	SE	DF	t value	p value
Intercept	0.687	0.317	110	2.169	0.032
Fishing effort	−0.535	0.176	110	−3.046	0.003
Location	0.174	0.062	110	2.793	0.006
Inshore	−0.829	0.321	40	−2.582	0.014
Midshore	−0.974	0.331	40	−2.944	0.005
Offshore	−0.862	0.346	40	−2.491	0.017
Effort:Inshore	0.544	0.201	110	2.71	0.008
Effort:Midshore	0.431	0.184	110	2.342	0.021
Effort:offshore	0.669	0.181	110	3.69	<0.001

Additional variables are demersal fish assemblage class (East, Inshore, Midshore, Offshore) and Location (latitude + longitude).

**Figure 6:**
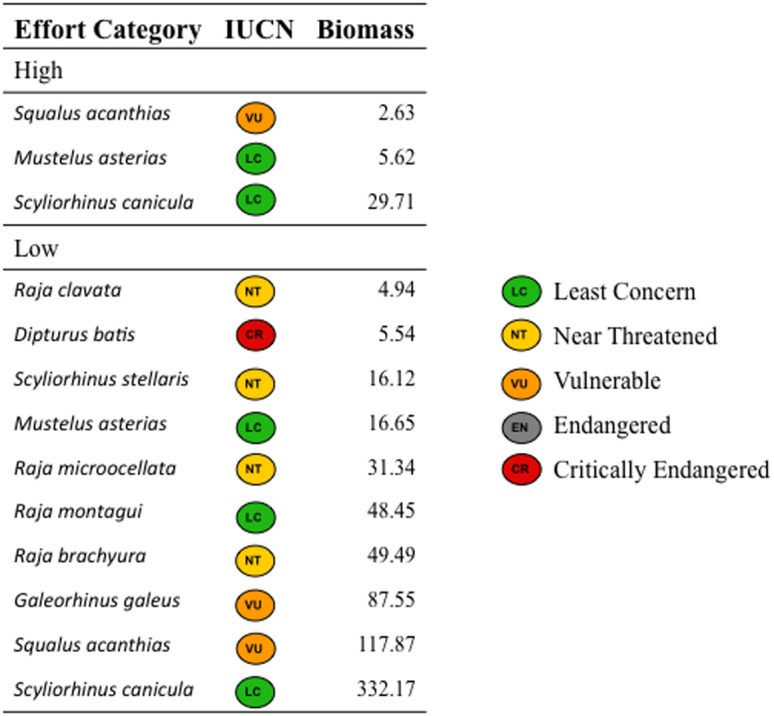
Standardised biomass (kg h^−1^) and species composition of elasmobranchs in survey hauls (2006–2011) at sampling sites in the upper (High) and lower (Low) 10% of the observed eastern Celtic Sea fishing effort (h y^−1^) range. IUCN status of each species is given.

There was a distinct area in the NE Celtic Sea where low fishing effort overlapped closely with greater elasmobranch biomass and species richness (Figure 5; Figure 6). This area showed moderate LPUE for commercial species, and was closer to port than other much more heavily fished areas of the Celtic Sea (Figure 5). However, fishermen indicated that the seabed in much of the area comprised highly dynamic sand features that made trawling inefficient and unpredictable. We therefore suggest that hypothesis (3: risk of losing gear due to rough or unpredictable seabed) likely best explains the low effort area of the Celtic Sea that now represents a *de facto* elasmobranch refuge.

## Discussion

The spatial distribution of fishing effort is often very uneven [12] and can remain stable over time [10]. In the NE Celtic Sea, this creates areas where annual fishing effort within a 20 km radius of IGFS survey sampling sites is consistently <3.0 h km^−2^. We find that these areas have many more elasmobranch species and greater elasmobranch biomass than geographically proximate heavily-fished areas. Our results suggest that heterogeneity in effort may create *de facto* refugia for Celtic Sea elasmobranchs, provided this mosaic of fishing effort distribution remains stable through time.

The distribution of elasmobranchs in the NE Atlantic shows broad patterns that are most likely driven by environmental parameters at regional scales (100 s km) [37]. Many elasmobranch species also respond to local habitat characteristics such as substratum type [47], [48] and depth [49], [50]. In this context, it might be suggested that remaining (relatively) high biomass patches just reflect areas where high quality habitat supported greatest elasmobranch biomass prior to fishing. Populations depleted by spatially homogenous fishing mortality would likely contract spatially into to such optimal areas [51]. However, we found that both fishing effort and habitat/environmental descriptors were retained as important explanatory variables in models of elasmobranch biomass. This suggests that *de facto* elasmobranch refugia may occur when low commercial fishing effort overlaps with favourable habitat.

Anecdotal information from fishers suggested that shifting sandy seabed in parts of the refuge area makes trawling difficult and hence uneconomic under the current management regime. The environment may thus impart some degree of on-going natural protection from fishing. However, some commercial fishing does occur in the area and LPUE can be quite high. This existing effort means that changes in the present fisheries management regime in the Celtic Sea (e.g., introduction of MPAs for other reasons) could displace the distribution of effort into this area [52] and perhaps quickly impair its value as an elasmobranch refuge. Historical data (noted by [53]) on fisheries discards of skates indicates that these species were previously abundant in areas proximate to the Celtic Sea refuge, from where they now have been almost extirpated.

Further work is required to understand how this *de facto* refuge functions to sustain elasmobranch biomass and species richness. Protection of nursery areas has been considered important in management of shark populations [54], [55]. Many shark species have distinct nursery areas, typically in nearshore areas [56]. Juveniles are often sedentary [57] meaning that they are likely to remain close to their natal area. Juveniles of *Raja clavata* can show strong site fidelity [58], and Frisk *et al*. [59] found that enhanced juvenile survival could help recovery of exploited skates. Notably, an analysis of long-term fisheries survey data (1967–2002) around the British Isles identified the area of greatest elasmobranch biomass observed in the current study as being important to juvenile *Rajids*, and also found that juveniles of the critically endangered *D. Batis* were only found in the Celtic Sea [60].

In contrast, recent evidence suggests that protection of adults may be a more effective elasmobranch conservation strategy than focusing on nursery grounds (see review in [55]). This is because deterministic stock/recruitment relationships mean that the contribution of juveniles to population growth rate is low compared to that of sub-adults and mature adults (e.g., [21]). For example, modelling suggests that a 3-season closure would protect Thames Estuary thornback ray from fishing pressure, but predominately by conserving larger size-classes [61]. Adults of some elasmobranch species, e.g. *D. batis*, are highly sedentary [62] and don't move out of low-effort areas where they receive some protection from fishing. Other ray populations can also benefit from MPAs, although the effect varies with species life history [63]. If protection of adult elasmobranchs is the optimal conservation strategy, then the discovery of a Celtic Sea refuge for at least ten elasmobranch species becomes even more important. Populations in this refuge, if protected, could possibly act as a source that would help sustain recruitment of these species in the Celtic Seas region.

The legal and management cost of establishing an MPA can be significant [64]. However, the key restrictions on the success of MPAs include negative cultural [65], political [66] and economic [67] impacts, and displacement of effort to other areas [68]. The Celtic Sea refuge area currently identified consistently receives very little effort and hence these problems may be limited because MPAs sited with reference to existing effort patterns are typically relatively effective [36]. At the most pragmatic level, such an MPA would protect an area for which survey data records greater elasmobranch abundance and species richness than anywhere else in the region. Annual fisheries displacement by the MPA could be less than three hours per km^2^.
